# The effect of sulfur baths on hemorheological properties of blood in patients with osteoarthritis

**DOI:** 10.1038/s41598-023-35264-8

**Published:** 2023-05-17

**Authors:** Aneta Teległów, Joanna Seremak, Joanna Golec, Jakub Marchewka, Piotr Golec, Urszula Marchewka, Marcin Maciejczyk, Edward Golec

**Affiliations:** 1 Department of Health Promotion, Institute of Basic Sciences, University of Physical Education in Krakow, 31-571 Kraków, Poland; 2 Solec Zdrój Health Resort, 28-131 Solec-Zdrój, Poland; 3 Institute of Clinical Rehabilitation, University of Physical Education in Krakow, 31-571 Kraków, Poland; 4 Individual Healthcare Centre, Kraków, Poland; 5 5th Military Clinical Hospital, 30-901 Kraków, Poland; 6 Institute of Biomedical Sciences, University of Physical Education in Krakow, 31-571 Kraków, Poland

**Keywords:** Diseases, Rheumatology

## Abstract

Balneotherapy is an effective treatment method in various diseases and commonly used treatment modality among patients with musculoskeletal disorders. Sulfur baths are known for healing properties however effect on rheological properties is unstudied. Thus the aim of our study was to determine the effect of sulfur balneotherapy on hemorheological blood indices. A total of 48 patients with osteoarthritis were enrolled to the study. Blood samples were collected twice, before and after 3-week time period. We evaluated complete blood count, fibrinogen, hs-CRP and blood rheology parameters such as elongation index (EI), half-time of total aggregation (T_1/2_) and aggregation index (AI) analyzed with the Lorrca Maxis. Mean age of studied cohort was 67 ± 5 years. After sulfur baths WBC count was significantly decreased is studied group (*p* = 0.021) as well as neutrophile count (*p* = 0.036). Red blood cell EIs were statistically higher after sulfur baths in shear stress ranging from 8.24 to 60.30 Pa. T_1/2_ was significantly higher (*p* = 0.031) and AI lower (*p* = 0.003) compared to baseline. No significant changes in fibrinogen and hs-CRP were observed. It is the first study that evaluate effect of sulfur balneotherapy on rheologic properties of blood. Sulfur water baths may improve erythrocyte deformability and aggregation parameters.

## Introduction

Balneotherapy is commonly used as a part of a treatment modality among patients with musculoskeletal disorders and has been found to be effective in the therapy of various disorders^1,2^. The mechanism of abovementioned therapy is unclear. It was proved that sulfur baths reduces tonicity, pain and joint swelling that partially is caused by increased sodium excretion, which stimulates renal diuresis that improve joints mobility^3,4^. According to Lengwant et al. groups treated with both physiotherapy and sulfur-salt water baths had significantly better outcomes in comparison to groups that were only treated with similar physical therapy, without baths. Balneotherapy procedures were found to have a significant impact on improvement of the joint function, the gait efficiency and kinematics. Moreover, balneotherapy was proved to have a meaningful effect on alleviation of pain and improvement in the quality of life^5,6^.

The most important natural resource of the Solec Zdrój Health Resort (SZHR) is healing sulfide water, considered the most effective in Poland. Due to its composition, the sulfide water in the SZHR is not subject to any chemical amelioration. The ideal parameters of the sulfide water in the spring, classified at the level of 137 mg H_2_S/l, means that the healing sulfide waters in the Solec Zdrój Health Resort are not diluted. Hydrogen sulfide in combination with sulfur, fluorine, iodine, bromine and boron affects the healing properties. The SZHR uses the private “Solec Shaft”. The health resort specializes in the treatment of diseases of the musculoskeletal system: degenerative, rheumatic, dermatological and neurological. The main active factor in the healing waters of the SZ is the sulfide ion, which is absorbed through the skin and reaches all tissues of the body through the blood^7,8^. Antioxidant effect H_2_S suppress reactive oxygen species and reactive nitrogen species and increases the expression of antioxidant enzymes^9^. H2S reduced the pro-inflammatory status induced by IL-1β in cultured human cellural chondrocytes. Moreover, H_2_S was found to have a protective effect against the degradation of the matrix in ex vivo experiments in osteoarthritis (OA) cartilage explants^10^. Recent studies in rat models shown that balneotherapy in sulfur-rich water diminishes the presence of oxidative damage markers, cartilage destruction and pain levels thus may be beneficial in non-pharmacological treatment of OA^11^. Karagülle et al.^12^ provided preliminary insights into the “biological truth” about natural H_2_S waters and their potential therapeutic role in balneology and health resort medicine.

To our knowledge the effect of sulfur water baths on rheological blood properties was not studied before. Thus the main aim of our study is to determine the effect of sulfide baths during a 3-week stay of patients in the SZHR on hemorheological blood indices, which include complete blood count, fibrinogen and deformability along with aggregation of red blood cells in patients with osteoarthritis.

## Materials and methods

### Participants

We enrolled 48 subjects (24 females and 24 males). Amongst them 35 participants (17 females and 18 males) underwent regular sulfur baths in Solec Zdrój Health Resort in Poland and 13 subjects (7 females and 6 males) was qualified to the control group without sulfur baths. Study inclusion criteria were: age between 60 and 80 years and diagnosis of osteoarthritis. Exclusion criteria included rheumatologic disorders, smoking, active infection and neoplasms. Participants were fully informed of the study details and expressed their written consent prior to taking part in the study. All the procedures complied with the Declaration of Helsinki and its further amendments^13^.

### Methods

The intervention group, consisting of 35 subjects, was qualified to fifteen sulfur baths in temperature 34–37 °C, each lasting 15 min. Sulfur baths were performed weekdays during 3-week stay in the Solec Zdrój Health Resort (Poland). Both study groups underwent similar standardized physical therapy sets consisting of kinesiotherapy, massage, manual therapy and laserotherapy. All interventions were supervised by medical doctor and nurse. Blood samples from all patients were collected at the beginning of treatment stay in the SZHR (baseline) and after a 3-week period (21 days). Qualified nurse collected twice 10 ml of fasting blood from the cubital vein into Vacuette EDTA K2 vacuum tubes. Hemorheological blood indices were determined in the Blood Physiology Laboratory of the University of Physical Education in Krakow and in the Diagnostyka S.A. laboratory in Krakow (Poland).

### Morphological and rheological assessments

Complete blood count was performed with an ADVIA 2120i Analyser (Siemens Healthineers, Erlangen, Germany) and involved white blood cell count (× 10^9^/L), neutrocyte count (× 10^9^/L), lymphocyte count (× 10^9^/L), monocyte count (× 10^9^/L), eosinocyte count (× 10^9^/L), basophil count (× 10^9^/L), red blood cell count (× 10^12^/L), hemoglobin concentration (g/dL), hematocrit (%), mean corpuscular volume (fL), mean corpuscular hemoglobin (pg), mean corpuscular hemoglobin concentration (g/dL), red blood cell distribution width (fL), platelet count (× 109/L), mean platelet volume (fL), procalcitonin concentration (%), and platelet distribution width (fL). Fibrinogen (g/L) was determined with a BCS Siemens coagulation analyser. Blood rheology parameters such as aggregation [aggregation index (%), amplitude and total extent of aggregation (arbitrary units) half-time of total aggregation (s)] and deformability of red blood cells (EI, elongation index) were tested with the Lorrca Maxsis (Lorrca, RR Mechatronics, The Netherlands) using method described by Hardeman and Baskurt^14,15^. The mean EI was plotted versus the corresponding shear stress of 0.30–60.00 Pa. The Lorrca is a functional Red Blood Cell analyzer capable of automated measurement of various red blood cell (RBC) phenomena by analysis of their rheological parameters. The technique accurately measures RBC deformability as a function of shear stress and aggregation RBC. C-reactive protein (CRP; an acutephase protein) concentration was evaluated with the immunonephelometric method, by using reagent kitsand a BN ProSpec nephelometer (Siemens Health). Coagulation parameters were determined using a BCS Siemens coagulation analyzer: INR, INR PT, APTT.

### Statistical analysis

Continuous variables are presented as mean ± standard deviation (SD) or median and interquartile range, depending on the normality of distribution. The normality of distribution was tested using the Shapiro–Wilk test. Qualitative variables were analyzed by enumerating the count and percentage occurrence of each value. Qualitative variables in groups were compared using the chi-squared test with Yates correction. To assess changes between the beginning and end of training we used *t* test for dependent samples, or Wilcoxon signed-rank test. For intergroup comparisons we used ANOVA or in case of not meeting its assumptions Kruskal–Wallis test. Calculations were performed using Statistica 13 (TIBCO Software Inc. USA) software. All *p* values are two-tailed, statistical significance was defined as *p* ≤ 0.05.

### Informed consent

Informed consent was obtained from all subjects involved in the study.

### Institutional review board statement

The study was conducted in accordance with the tenets of the Declaration of Helsinki and approved by the Ethics Committee of the Regional Medical Chamber in Krakow, Poland (Approval No. 212/KBL/OIL/2022).

## Results

The intervention group consisted of 35 subjects with mean age 67.7 ± 5.4 years; 17 females (49%) and 18 males (51%). The control group involved 13 subjects with mean age 66.2 ± 3.4 years; 7 females (54%) and 6 males (46%). Detailed group characteristics are presented in Table 1.

**Table 1:** Basic characteristics of the study groups.

Parameter	Sulfur bath group N = 35	Control goup N = 13	*p*
Age, year (mean ± SD)	67.7 ± 5.4	66.2 ± 3.4	> 0.05
Sex, female n (%)	17 (49%)	7 (46%)	> 0.05
Comorbidities			
Acute infection, n (%)	0 (0)	0 (0)	–
Diabetes mellitus, n (%)	5 (14%)	2 (15%)	> 0.05
Neoplasms, n (%)	0 (0)	0 (0)	–
Rheumatologic disorders, n (%)	0 (0)	0 (0)	–
Smoking, n (%)	0 (0)	0 (0)	–
Spondylolisthesis, n (%)	3 (9%)	1 (8%)	> 0.05
Urinary incontinence, n (%)	1 (3%)	0 (0)	> 0.05

*P*—for quantitative variables Mann–Whitney test, for qualitative variables chi-squared test or Fisher's exact test (*p* < 0.05).

*SD* standard deviation.

No statistically significant differences between studied groups at baseline measurements were found. We observed statistically significant differences between WBC, HGB, MCH, MPV, NEU parameters after sulfur baths. Detailed complete blood count results are presented in Table 2.

**Table 2:** Mean values (± SD—standard deviation) of the morphological parameters among health resort patients before and after treatment stay and in the control group.

Parameter	Baseline N = 35	After 3 weeks of sulfur bath N = 35	Controls N = 13	*P* (dependent)
WBC (10^9/l)	6.94 ± 2.46	6.31 ± 1.52	5.70 ± 1.18	0.021*
RBC (10^12/l)	4.66 ± 0.44	4.65 ± 0.43	4.57 ± 0.31	> 0.05
HGB (g/dl)	14.27 ± 1.10	14.14 ± 1.07	14.05 ± 0.92	0.020*
HCT (%)	42.28 ± 3.14	42.16 ± 2.90	41.28 ± 2.80	> 0.05
MCV (fl)	90.99 ± 4.50	90.89 ± 4.38	88.75 ± 4.63	> 0.05
MCH (pg)	30.71 ± 1.54	30.46 ± 1.55	30.77 ± 1.58	0.0003*
MCHC (g/dl)	33.77 ± 1.15	33.54 ± 1.20	33.68 ± 0.81	> 0.05
PLT (10^9/l)	246.97 ± 60.02	253.57 ± 59.90	226.31 ± 48.47	> 0.05
RDW-SD (fl)	44.11 ± 2.69	43.96 ± 3.04	44.06 ± 3.00	> 0.05
RDW-CV (%)	13.31 ± 0.71	13.25 ± 0.69	13.04 ± 0.92	> 0.05
PDW (fl)	13.40 ± 2.13	13.06 ± 2.18	14.05 ± 2.88	> 0.05
MPV (fl)	11.01 ± 0.99	10.78 ± 0.87	11.31 ± 1.25	0.016*
P-LCR (%)	33.37 ± 8.19	31.36 ± 7.24	35.49 ± 9.84	0.005*
PCT (%)	0.28 ± 0.07	0.28 ± 0.06	0.25 ± 0.05	> 0.05
NEU (10^9/l)	3.53 ± 1.31	3.18 ± 1.00	3.29 ± 0.60	0.036*
LYM (10^9/l)	2.52 ± 2.22	2.23 ± 1.16	2.05 ± 0.77	> 0.05
MONO (10^9/l)	0.61 ± 0.17	0.64 ± 0.19	0.57 ± 0.12	> 0.05
EOS (10^9/l)	0.22 ± 0.15	0.22 ± 0.17	0.15 ± 0.07	> 0.05
BASO (10^9/l)	0.04 ± 0.01	0.04 ± 0.02	0.04 ± 0.02	> 0.05
NEU (%)	51.78 ± 11.17	50.34 ± 8.96	53.02 ± 7.56	> 0.05
LYM (%)	34.83 ± 11.88	34.91 ± 9.83	35.22 ± 7.53	> 0.05
MONO (%)	9.32 ± 2.26	10.62 ± 4.09	8.39 ± 2.07	> 0.05
EOS (%)	3.39 ± 2.60	3.47 ± 2.57	2.63 ± 1.35	> 0.05
BASO (%)	0.68 ± 0.24	0.65 ± 0.30	0.73 ± 0.28	> 0.05

*Significant difference (*p* < 0.05).

We have not found statistically significant differences between analyzed groups in high sensivity C-reactive protein concentrations, fibrinogen levels and coagulation parameters as shown in Table 3.

**Table 3:** Mean values (± SD—standard deviation) or median (interquartile range) of the selected biochemical and blood coagulation parameters among health resort patients before and after treatment stay and in the control group.

Parameter	Baseline N = 35	After 3 weeks of sulfur bath N = 35	Controls N = 13	*P* (dependent)
hs-CRP (mg/l)	1.43 (0.67–2.32)	1.50 (0.79–3.48)	1.77 (0.48–1.98)	> 0.05
FIBR (g/l)	2.94 ± 0.64	2.93 ± 0.57	3.24 ± 0.63	> 0.05
INR	1.02 ± 0.38	0.98 ± 0.17	0.97 ± 0.13	> 0.05
INR PT (%)	102.32 ± 14.56	103.97 ± 11.79	104.77 ± 12.05	> 0.05
PT (s)	13.84 ± 1.60	10.67 ± 1.48	12.62 ± 1.17	> 0.05
APTT (s)	29.91 ± 3.94	29.74 ± 3.35	32.85 ± 11.85	> 0.05

*Significant difference (*p* < 0.05).

Assessment of red blood cells aggregation parameters in intervention group revealed statistically significant changes in half-time of total aggregation and aggregation index as presented in Table 4.

**Table 4:** Mean values (± SD—standard deviation) of red blood cells rheological parameters among health resort patients before and after treatment stay and in the control group.

Parameter	Baseline N = 35	After 3 weeks of sulfur bath N = 35	Controls N = 13	*P* (dependent)
AMP (au)	29.11 ± 8.66	31.51 ± 10.85	30.98 ± 5.30	> 0.05
T_1/2_ (s)	1.92 ± 0.64	2.38 ± 0.58	1.94 ± 0.99	0.031*
AI (%)	65.78 ± 8.38	61.00 ± 9.47	66.89 ± 7.88	0.003*

*Significant difference (*p* < 0.05).

**Figure 1:**
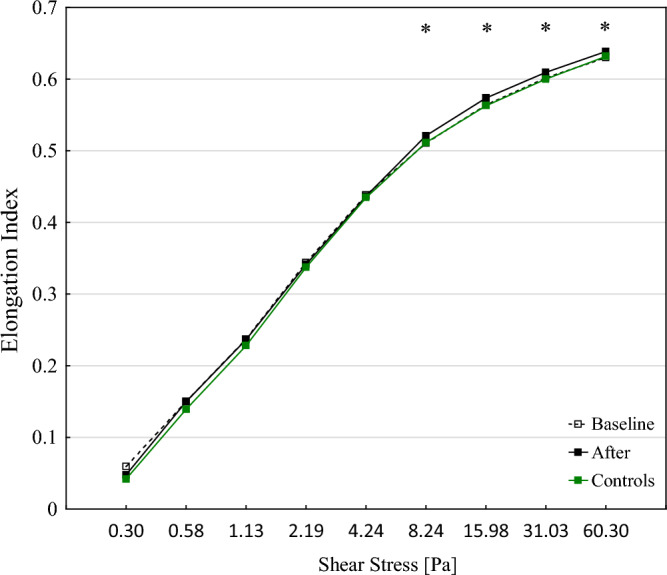
Elongation index (EI)–shear stress (SS) curves for RBC in studied groups. *p < 0.05 compared to baseline and controls.

Assessment of red blood cells deformability parameters in intervention group revealed statistically significant changes in Red blood cell EIs. Were statistically higher after sulfur baths in shear stress ranging from 8.24 to 60.30 Pa as shown in Fig. 1.

## Discussion

According to our knowledge, there are no clinical reports that address the effect of balneological therapies, especially sulfur water baths, on combined rheological and biochemical blood parameters in a comprehensive and standardized study.

We found that rheological parameters described by elongation index, which determine red blood cell length changes in relation to width while stretched, were improved after Solec Zdrój Health Resort treatment. Those results were significantly higher while using increased shear stresses such as 8.24, 15.98, 31.03 and 60.30 Pa. The deformability of red blood cells has an important role in their main function, which is the transportation of oxygen and carbon dioxide via blood circulation^16^. The average size of a single erythrocyte is 7–8 µm, while the diameter of capillaries is 3–5 µm which makes it necessary to deform them—the greater the better^17,18^. According to Kim et al. a slight decrease in RBC deformability causes a significant increase in microvascular flow resistance and blood viscosity. Moreover, reduced RBC deformability measured with EI was observed in particular diseases with capillary disorders such as sickle cell and cardiovascular diseases as well as diabetes mellitus and its complications^19–21^. Additionally, Gelmini et al.^22^ found that red blood cells deformability lowers with age which affects tissue oxygenation. Franzini et al.^23^ found that significant decrease in the deformability of red blood cells among older patients was connected with increased membrane cholesterol which affects membrane viscosity. The exact physiological mechanism that the sulfur bath induces on the hemorheological parameters is not known. H_2_S shows antioxidant properties, as it quenches reactive oxygen species (ROS) and reactive nitrogen species (RNS) and increases the expression of antioxidant enzymes by activating the transcription factor nuclear factor erythroid-derived 2-like 2 (Nrf-2)^9^. It is known that ROS cause vascular cell damage, the recruitment of inflammatory cells, lipid peroxidation, collectively leading to vascular remodeling^24^. Taking abovementioned into consideration it could be hypothesized that anti-oxidant properties of sulfur baths may improve hemorheological blood properties. Moreover, hydrogen sulphide forms fat-soluble polysulphides in the skin, that allows to enter the bloodstream through the capillaries. It is hypothesized that sulfur baths positive effect on the rheological properties of blood might be connected with endothelial cells, so they can regulate nitric oxide (NO) discharge, which is strong vasodilatory and anti-inflammatory signaling molecule^25^. NO synthesis in endothelial cells is controlled by many factors, including the shear forces acting on the vessel walls, which in turn are determined by the flow and viscosity of the blood in the peripheral part of the vessel^26,27^. In small-diameter capillaries, erythrocytes flow in one row, using the maximum capacity of their deformability. Increased blood flow in the small arteries of fingers was reported in patients with rheumatoid arthritis after bathing in sulphide water^5^. This is in line with our study where we found increased erythrocytes deformability in shear stresses including 8.24, 15.98, 31.03 and 60.30 Pa.

It is important not only to evaluate the red blood cells deformability index but also RBC aggregation parameters. Interestingly we found that the time required for half maximal change in aggregation signal (T_1/2_) of RBC was significantly lower among patients treated in health resort with sulfur water baths. It was comparable with previous study among triathlon subjects^22^. Factors affecting the formation of red blood cells aggregates may be divided into two groups. The first group consists of external factors, includes the level of plasma proteins (fibrinogen, lipo-proteins, macroglobulins, immunoglobulins), shear forces and hematocrit. The second group consists of internal factors, such as the shape of erythrocytes, their deformability and the properties of the cell membrane^28^. Moreover, patients treated in health resort had significantly lower results of the extent of red blood cells aggregation described with aggregation index (AI). Similarly to deformability, the aggregation of red blood cells has an important impact on their main function—transportation, however the lower the aggregation index the better and the higher T_1/2_ the better as well. We did not find any significant differences in red blood cells count between study groups, similar results were found by Galvez^29^. According to Baskurt et al. rheological properties of blood mainly results from the properties of red blood cells, which strongly modify blood flow in blood vessels, but also from the properties of plasma, including proteins contained in it. The presence of high molecular weight proteins, such as fibrinogen, lipoproteins and globulins (especially α2-macroglobulin and immunoglobulins), increase plasma viscosity and thus increase blood viscosity^30^.

We found that the level of white blood cells (WBC) significantly lowered among patients with osteoarthritis treated in SZ health resort. Speaking of granulocytes, which are the types of WBC, the biggest difference was seen in neutrophiles, which number has also significantly decreased. Such results may suggest that treatment in sulfur water in health resort has positive impact on chronic low-grade inflammation process as presented by Xu et al.^31^

However, we did not observe any significant differences in high sensitive C-reactive protein concentrations. Our results are in opposition to those presented by Olah, in which CRP significantly decreased, nevertheless, second blood specimen collection time was different between studies, 3 weeks versus 3 months accordingly^25,26^. In some cases, the rheological properties of blood depend on fibrinogen. Its lower level affects the initiation of red blood cell aggregation, which leads to increased blood viscosity.

Fibrinogen is a 340 kDa glycoprotein synthesized in the liver, with plasma concentrations of approximately 150–400 mg/dl; is a protein involved in blood coagulation and hemostasis, it is also involved in inflammation and tissue repair processes. Fibrinogen facilitates platelet aggregation by binding the glycoprotein IIb/IIIa receptor and forming a fibrin monomer that rapidly polymerizes to form a clot^32,33^. According to Sen et al. plasma fibrinogen levels increase 2- to 3-times during the inflammatory response, which causes cell aggregation and increases blood viscosity^34^. The rheological properties of blood depend to a large extent on the fibrinogen concentration. Its elevated level leads to the increased red blood cell aggregation, which result in increased blood viscosity. However, we have not found statistically significant differences between analyzed groups in fibrinogen levels and coagulation parameters.

Moreover, we found that the mean hemoglobin levels among patients treated in health resort significantly decreased after 3 weeks. It may be relative, due to increased thirstiness after bathing in sulfide waters that induces overhydration. Abovementioned situation results in fluctuation of blood pressure and depending on the speed of this reaction and on the adaptive capacity of the human body, the heart rate may also alter^35^.

### Limitations

The study has limitations. Firstly, the control group was smaller than intervention one however there were no statistically significant differences between these groups in sex ratio, age and other baseline parameters. Furthermore, study design was prospective. Secondly, we have performed measurement at baseline and after 3-week time that reflects course of sulfur balneotherapy. Average RBC lifespan in normal individual is 115 days^36^. Thus, when taking it into account, effects on red blood cell hemorheological properties are probably even underestimated. Thirdly, along with the laboratory parameters functional assessment with quality of life evaluation could be performed, but our study intended to assess biochemical and rheological background of sulfur balneotherapy.

## Conclusions

Osteoarthritis is a widespread disease causing pain and inflammation that limits mobility and functionality^37,38^. Despite high prevalence of osteoarthritis, the effective treatment is undetermined. Sulfur water baths may improve erythrocyte deformability and aggregation parameters in patients with osteoarthritis along with reducing of neutrophile levels. The efficacy of balneotherapy, especially sulfur water baths should be the subject of further research.

## Data Availability

The datasets used and/or analysed during the current study are available from the corresponding author on reasonable request.

## Abbreviations

SZHR: Solec Zdrój Health Resort
AI: Aggregation index
EI: Elongation index
WBC: White blood cells
RBC: Red blood cells
LYM: Lymphocytes concentration
ON: Monocytes concentration
NEU: Neutrophil concentration
EOS: Eosinophil concentration
BASO: Basophil concentration
RBC: Red blood cells
HGB: Hemoglobin
HCT: Hematocrit
MCV: Mean corpuscular volume
MCH: Mean corpuscular hemoglobin
MCHC: Mean corpuscular hemoglobin concentration
RDW-CV: Red blood cell distribution width coefficient of variation
RDW-SD: Red blood cell distribution width standard deviation
PLT: Platelet concentration
PDW: Platelet distribution width
P-LCR: Platelet-large cell ratio
PCT: Plateletcrit
INR: International normalized ratio
PT: Prothrombin time
INR–PT: International normalized ratio–prothrombin time
APTT: Activated partial thromboplastin time
AMP: Erythrocyte aggregation amplitude
T_1/2_: Half-time of total red blood cell aggregation
AI: Aggregation index
EI: Elongation index
